# No effect of exogenous melatonin on development of cryopreserved metaphase II oocytes in mouse

**DOI:** 10.1186/s40104-015-0041-0

**Published:** 2015-09-14

**Authors:** Wei Li, Keren Cheng, Yue Zhang, Qinggang Meng, Shi’en Zhu, Guangbin Zhou

**Affiliations:** Institute of Animal Genetics and Breeding, College of Animal Science and Technology, Sichuan Agricultural University (Chengdu Campus), Wenjiang, 611130 P.R. China; Institute of Animal Genetics and Breeding, College of Animal Science and Technology, China Agricultural University, Beijing, 100193 P.R. China; Department of Animal, Dairy, and Veterinary Sciences, Utah State University, Logan, Utah USA; Nanjing Biomedical Research Institute of Nanjing University, Nanjing, 210089 P.R. China

**Keywords:** Gene expression, Melationin, Mouse oocyte, Parthenogenetic activation, vitrificantion

## Abstract

**Background:**

This study was conducted to investigate effect of exogenous melatonin on the development of mouse mature oocytes after cryopreservation.

**Results:**

First, mouse metaphase II (MII) oocytes were vitrified in the open-pulled straws (OPS). After warming, they were cultured for 1 h in M_2_ medium containing melatonin at different concentrations (0, 10^−9^, 10^−7^, 10^−5^, 10^−3^ mol/L). Then the oocytes were used to detect reactive oxygen species (ROS) and glutathione (GSH) levels (fluorescence microscopy), and the developmental potential after parthenogenetic activation. The experimental results showed that the ROS level and cleavage rate in 10^−3^ mol/L melatonin group was significantly lower than that in melatonin-free group (control). The GSH levels and blastocyst rates in all melatonin-treated groups were similar to that in control. Based on the above results, we detected the expression of gene *Hsp90aa1*, *Hsf1*, *Hspa1b*, *Nrf2* and *Bcl-x1* with qRT-PCR in oocytes treated with 10^−7^, or 10^−3^ mol/L melatonin and untreated control. After warming and culture for 1 h, the oocytes showed higher *Hsp90aa1* expression in 10^−7^ mol/L melatonin-treated group than in the control (*P* < 0.05); the *Hsf1*, *Hsp90aa1* and *Bcl-x1* expression were significantly decreased in 10^−3^ mol/L melatonin-treated group when compared to the control. Based on the above results and previous research, we detected the development of vitrified-warmed oocytes treated with either 10^−7^ or 0 mol/L melatonin by *in vitro* fertilization. No difference was observed between them.

**Conclusions:**

Our results indicate that the supplementation of melatonin (10^−9^ to 10^−3^ mol/L) in culture medium and incubation for 1 h did not improve the subsequent developmental potential of vitrified-warmed mouse MII oocytes, even if there were alteration in gene expression.

## Background

Free radicals and reactive oxygen species (ROS), generated as a part of normal cellular metabolism and as a consequence of exogenous administered molecules [1], play an important role as second messengers in cellular functions through activation of cell signaling cascades, such as those involving in mitogen-activated protein kinases and regulation of transcription factors. Excessive ROS, however, are highly reactive with complex cellular molecules (proteins, lipids, and DNA) and may change their functions [2]. This may lead to serious consequences, for instance, enzymatic inactivation, DNA fragmentation, and ultimately cell death [3–6]. Glutathione (GSH) is a major antioxidant acting as a free radical scavenger that protects the cell from ROS. The balance between ROS and GSH had been considered in controlling the oocyte maturation and the normal development of zygotes [7, 8]. During cryopreservation, oocytes are particularly vulnerable to oxidative stress because of the high level of lipid, generating large amount of ROS [9], which influence the balance between the oxidation–reduction reactions and the intracellular antioxidative system. An imbalance in this system in the favor of oxidation significantly reduced cell viability [10].

Transcription factor Nrf2 (nuclear factor-erythroid 2 p45-related factor 2) participates in the transcription regulation of enzyme which was involved in the GSH synthesis metabolism [11], consequently regulating the balance of ROS/GSH [12]. Transcription factor Hsf1 (heat shock factor 1) was involved in both the regulation of the balance of ROS/GSH and the transcription of *Hsp90* and *Hsp70*. Heat shock proteins (HSP), a set of proteins generated under stress, were associated with RNA processing, RNP assembly, and chromatin remodeling [13], and among them, maternal Hsp90 and Hsp70 were required for the embryonic development [14–17]. Transcription factors Hsf1 and Nrf2 engaged in crosstalk for cytoprotection by sharing overlapping transcriptional targets, such as HSP70 [11]. After oocytes cryopreservation, the expression of *Hsp70* [18] and *Hsp90β* [19] was significantly decreased, potentially influencing their subsequent development potential.

Melatonin (N-acetyl-5-methoxytryptamine), a derivative of tryptophan mainly produced in the pineal gland of vertebrates [20, 21], is a potent free radical scavenger and antioxidant [22, 23]. Melatonin and its metabolites could directly scavenge ROS, stimulate antioxidative enzymes, increase the levels of GSH, inhibit the pro-oxidative enzymes in cells and organs [24–26], and promote the expression of antiapoptotic gene *Bcl-xl* [27]. When melatonin was added to semen extender or culture medium, the sperm viability, oocyte competence and blastocyst development *in vitro* were significantly improved (reviewed by [23]). However, it is still unclear whether or not the oocyte development could be improved by the addition of melatonin to the medium for *in vitro* culture of vitrified-warmed mouse metaphase II (MII) oocytes.

Therefore, in this study, we investigated the effect of melatonin on developmental potential of vitrified mouse oocytes, including detecting ROS and GSH levels, expressions of apoptosis related genes (*Hsp90aa1, Hsf1, Hspa1b, Nrf2* and *Bcl-x1*), subsequent embryonic development after parthenogenetic activation and *in vitro* fertilization.

## Materials and methods

Unless otherwise stated, all chemicals were purchased from Sigma-Aldrich (St. Louis, MO, USA). All animals were maintained and handled in accordance with the requirements of the Institutional Animal Care and Use Committee of the China Agricultural University.

### Oocyte collection

Outbred female Kun Ming mice (different from the typical inbred strains [28]) (China Experimental Animal Center of Military Medical Sciences, China) aged 6 wk were kept in a room with the temperature controlled at 20–22 °C under a 14:10 light/dark cycle (light on at 06:00 h). After a week of acclimation, female mice were induced to superovulate by an intraperitoneal injection of 10 IU equine chorionic gonadotropin initially, and 48 h later, 10 IU human chorionic gonadotropin (hCG) was injected to trigger ovulation, as described previously [29]. Cumulus–oocyte complexes were collected from oviducts at 14 h after hCG treatment and recovered in M_2_ medium [30] supplemented with 3 mg/mL bovine serum albumin. Cumulus cells were dispersed with 300 IU/mL hyaluronidase.

### Vitrification and warming of oocytes

The open-pulled straws (OPS) were made according to the method as described previously [31, 32] with some modifications. Briefly, the straws (250 mL; IMV, L’Aigle, France) were heat-softened and pulled manually to get a straw of approximately 2 to 3 cm in length, 0.10 mm in inner diameter, and 0.15 mm in outer diameter.

Oocytes were vitrified using an OPS method. Oocytes were first equilibrated in 10 % ethylene glycol (EG) + 10 % dimethyl sulfoxide (DMSO) in Dulbecco phosphate-buffered saline (DPBS) containing 20 % fetal bovine serum (FBS; Hyclone; Gibco BRL, Paisley, Scotland, UK) for 30 s, then loaded into the narrow end of OPS with EDFS30 solution which consisted of DPBS medium containing 300 g/L Ficoll, 0.5 mol/L sucrose, and 20 % FBS, 15 % (v/v) EG and 15 % (v/v) DMSO, for 25 s. Finally, the straws containing oocytes (10 oocytes per OPS) were plunged into liquid nitrogen. When warming, oocytes were rinsed in 0.5 mol/L sucrose for 5 min, then washed 3 times in M_2_ medium and incubated in a CO_2_ incubator for 1 h in M_2_ medium with different concentration of melatonin. All manipulations were performed at 37 °C on a warming stage fixed on the stereomicroscope, and the ambient atmosphere was air-conditioned at a temperature of 25 ± 0.5 °C. Oocytes were pooled and randomly distributed to each group.

### Measurement of intracellular reactive oxygen species and glutathione levels

Mouse MII oocytes were sampled to determine the intracellular ROS and GSH levels according to the method described in previous study [33]. To measure intracellular ROS level, more than 15 oocytes from each treatment group were incubated (in the dark) in M_2_ supplemented with 1 mmol/L 20,70-dichlorodihydrofluorescein diacetate (H2DCFDA) for 20 min at 37 °C, washed three times with DPBS containing 0.1 % (w/v) polyvinyl alcohol, and then placed into 50 mL droplets. The fluorescence was measured under an epifluorescence microscope with a filter at 460-nm excitation, and fluorescence images were recorded as TIFF files using a cooled CCD camera (DP72, Olympus, Tokyo, Japan). The recorded fluorescence intensities were quantified by EZ-C1 Free Viewer software (Nikon, Tokyo, Japan). The level of GSH in each oocyte was measured with 10 μmol/L 4-chloromethyl-6.8-difluoro-7-hydroxycoumarin (Cell-Tracker Blue) with a filter at 370-nm excitation. The experimental procedure was the same as the ROS measurement described above.

### Oocyte activation and embryo culture

All treated oocytes were allowed to recover in a CO_2_ incubator for 1 h before activation. The activation medium used was Ca^2+^-free human tubal fluid (HTF) [34] supplemented with 10 mmol/L SrCl_2_ [35]. After being washed thrice in activation medium, oocytes were incubated first in activation medium for 2.5 h and then in regular HTF without SrCl_2_ for 3.5 h at 37.5 °C in a humidified atmosphere with 5 % CO_2_ in air. Both the activation medium and HTF for subsequent short culture of oocytes were supplemented with 2 μg/mL cytochalasin D. Six h after the onset of activation, oocytes were removed from the medium and cultured in KSOM-AA (simplex optimized medium contained K ions supplemented with amino acids) medium [36] (Millipore) for 4 d. Embryos at the two cell and blastocyst stages were examined and recorded at 24, and 96 h after start of culture in KSOM-AA medium, respectively.

### Quantitative Polymerase Chain Reaction(Q-PCR)

Total RNA was isolated from 50 mouse oocytes for each group by using Trizol reagent (Invitrogen, Carlsbad, CA). The RNA was reverse transcribed into complementary DNA(cDNA) using the High Capacity cDNA Reverse Transcription (RT) kit (Applied Biosystems, CA, USA); then, the cDNA was quantified by Q-PCR using a SYBR PrimeScript RT-PCR Kit (TaKaRa, Dalian, China) on a CFX96 Real-Time PCR Detection System (Bio-Rad, CA, USA) under standard conditions. The cycle threshold (Ct) value used to calculate the relative expression was the average of three replicates and was normalized against that of the reference gene (GAPDH). The primer information was summarized in Table 1. The mRNA expression levels were calculated using the 2^-△△Ct^ method [37].

**Table 1:** PCR primers used for SYBR green Q-PCR analysis

Gene name	Assay ID	Primer Seq (5'→3')	Product length, bp	Tm, °C
*Hsp70 (Hspa1b)*	NM_010478	F:TGTTCCAGTAGCCTGGGAAG	165	58
		R:CCACAAAACCTTAACATGGACA		
*Hsp90 (Hsp90aa1)*	NM_010480	F:AAGGCAGAGGCTGACAAGA	212	58
		R:AGGGGAGGCATTTCTTCAGT		
*Nrf2 (Nfe2l2)*	NM_010902.3	F:CAGTGCTCCTATGCGTGAA	109	58
		R:GCGGCTTGAATGTTTGTC		
*Hsf1*	NM_008296N.2	F:GCTCTGGACCCATAATCTC	122	58
		R:CTCTTGCTTGACACGGAC		
*Bcl-xl*	NM_001289716.1	F:GACAAGGAGATGCAGGTATTGG	124	58
		R:TCCCGTAGAGATCCACAAAAGT		
*GAPDH*	NM_008084.3	F:CATGGCCTTCCGTGTTCCTA	104	58
		R:GCCTGCTTACCACCTTCTT		

### *In vitro* fertilization (IVF)

The fresh and vitrified-warmed oocytes were first individually placed into 70 μL drops of human tubal fluid (HTF) medium (Millipore) under mineral oil, then 10 μL of capacitated sperm, which had been incubated for 1–1.5 h in HTF medium in a CO_2_ incubator, was added to the oocytes. The final concentration was 2.0-6.0 × 10^6^ sperm/mL. Five h after IVF, the oocytes were removed from the fertilization drops, washed in KSOM-AA medium (Millipore) 3 times, and cultured in 70 μL drops of KSOM-AA medium. Embryos at the two-cell and blastocyst stages were examined and recorded at 24, and 96 h after start of culture in KSOM-AA medium, respectively.

### Statistical analysis

Statistical analysis was conducted by one-way ANOVA followed by Duncan’s test using SPSS statistical software (IBM, IL, USA). Data were expressed as the mean ± standard error, and ***P*** < 0.05 was considered significant.

## Results

### Effect of melatonin on redox state in vitrified-warmed mouse mature oocytes

**Fig. 1:**
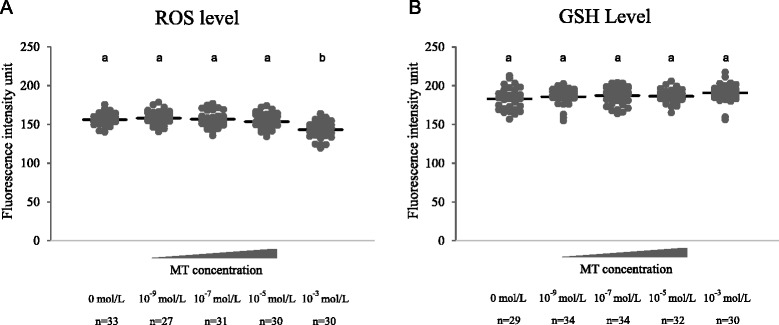
Effects of melatonin on intracellular levels of reactive oxygen species (ROS) and glutathione (GSH) in vitrified-warmed mouse oocytes. Fluorescence intensities were correlated with intracellular levels of ROS and GSH. Number of Oocytes in each group (n). **a** and **b** denote significant differences (*P* < 0.05)

After warming, mouse MII oocytes were cultured for 1 h in M_2_ medium containing different concentrations (0, 10^−9^, 10^−7^, 10^−5^, 10^−3^ mol/L) of melatonin, respectively. Then the oocytes were used for detection of ROS and GSH levels. As shown in Fig. 1, the ROS level was lower (*P* < 0.05) in 10^−3^ mol/L melatonin-treated group than in melatonin-free group (control), and the GSH level in melatonin-treated groups showed no significant difference (*P* > 0.05) when compared with control group.

### Effect of melatonin on parthenogenetic development of vitrified-warmed mouse metaphase II oocytes

As shown in Table 2, when vitrified-warmed mouse MII oocytes were cultured for 1 h in M_2_ medium with different concentrations (0, 10^−9^, 10^−7^, 10^−5^, 10^−3^ mol/L) of melatonin, respectively, followed by parthenogenetic activation, the cleavage rate decreased significantly in 10^−3^ mol/L melatonin-treated group when compared with control group, but the blastocyst rate in all melatonin-treated groups was similar to (*P* > 0.05) that in control group.

**Table 2:** Parthenogenetic development of vitrified-warmed mouse MII oocytes after melatonin treatment

Group (melatonin treatment)	Total No. of oocytes examined	No. of oocytes survived	No. of oocytes developed to	
			2-cell (%)^a^	Blastocyst (%)^a^
0 mol/L	60	51	41(80.39 ± 5.19)^b^	27(52.94 ± 5.88)^b^
10^−9^ mol/L	120	97	73(81.31 ± 3.60)^b^	47(55.04 ± 3.80)^b^
10^−7^ mol/L	100	88	67(79.95 ± 4.05)^b^	40(42.36 ± 6.08)^b^
10^−5^ mol/L	100	84	57(74.24 ± 6.73)^b^	41(54.58 ± 6.11)^b^
10^−3^ mol/L	100	88	50(54.84 ± 7.92)^c^	29(47.22 ± 2.78)^b^

1,^.a^ Number of 2-cell or blastocyst/Number of oocytes survived

2, Percentage data are presented as mean ± SEM from at least 3 replicates b and c, denote significant differences (*P* < 0.05)

3, Melatonin treatment: the vitrified-warmed mouse MII oocytes were cultured for 1 h in M_2_ medium with different concentrations (0, 10^−9^, 10^−7^, 10^−5^, 10^−3^mol/L) of melatonin, respectively, then they were used for parthenogenetic activation

### Effect of melatonin on genes expression in vitrified-warmed mouse metaphase II oocytes

**Fig. 2:**
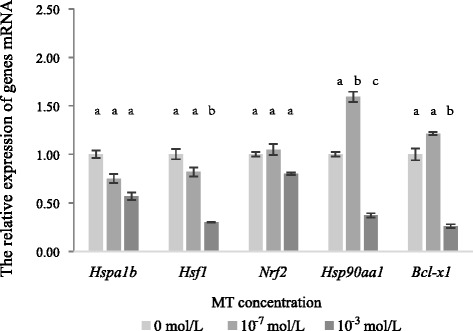
Effect of melatonin on genes expression of mRNA in vitrified-warmed mouse oocytes. The relative expression level of mRNA were determined by the 2^-ΔΔCT^ method and normalized against GAPDH. All data are mean ± SEM from 3 replicates. a, b and c, denote significant differences (***P*** < 0.05)

As shown in Fig. 2, when vitrified-warmed mouse MII oocytes were cultured for 1 h in M_2_ medium with different concentrations (0, 10^−7^, 10^−3^ mol/L) of melatonin, respectively, the expressions of *Hsp90aa1*, *Hsf1*, *Hspa1b*, *Nrf2* and *Bcl-x1* were decreased in the 10^−3^ mol/L melatonin-treated group when compared with the other two groups. But the expressions of *Hsf1*, *Hsp90aa1* and *Bcl-x1* in the 10^*−3*^ mol/L melatonin-treated group were lower than those in the melatonin-free group (*P* < 0.05). Compared with the melatonin-free group, the 10^−7^ mol/L melatonin-treated group showed decreased expressions in genes *Hsf1* and *Hspa1b*, increased expression in genes *Hsp90aa1*, *Nrf2* and *Bcl-x1*, and significantly increased (*P* < 0.05) expression in gene *Hsp90aa1* .

### Effect of melatonin on subsequent embryonic development after IVF

As shown in Table 3, when vitrified-warmed mouse MII oocytes were cultured for 1 h in M_2_ medium with different concentrations (0 and 10^−7^ mol/L) of melatonin, respectively, followed by IVF. The fresh mouse MII oocytes were used as control. Either the cleavage or the blastocyst rates in both the melatonin-treated and melatonin-free groups were similar, but they were significantly lower (*P* < 0.05) when compared with the fresh control group.

**Table 3:** The subsequent embryonic development of vitrified-warmed mouse MII oocytes treated with melatonin followed by IVF

Group	Melatonin concentration	No of oocytes used for IVF	No. of oocytes developed to	
			2-cell(%)^a^	Blastocyst(%)^a^
Fresh	0 mol/L	60	57(93.55 ± 2.54)^b^	51(84.78 ± 0.36)^b^
Vitrified	0 mol/L	58	35(60.53 ± 5.48)^c^	25(43.51 ± 12.61)^c^
Vitrified	10^−7^ mol/L	59	33(55.39 ± 8.02)^c^	23(36.93 ± 6.15)^c^

1,^a^ Number of 2-cell or blastocyst/Number of oocytes used for *in vitro* fertilization (IVF)

2, Percentage data are presented as mean ± SEM from at least 3 replicates.^b^ and^c^, denote significant differences (*P* < 0.05)

3, Melatonin treatment: the vitrified-warmed mouse MII oocytes were cultured for 1 h in M_2_ medium with different concentrations (0, 10^−7^ mol/L) of melatonin, respectively, then they were used for IVF. The fresh mouse MII oocytes were used as control

## Discussion

Mammalian oocytes with complicated subcellular structure are sensitive to the temperature and osmotic pressure changes [38]. During cryopreservation, changes could occur in the microenvironment of the oocytes, such as the formation and release of large amounts of ROS [9], consequently influencing the quality of oocytes [39]. The excessive ROS production due to oocyte cryopreservation could disturb the balance between the oxidation-reduction reaction and the antioxidant system, and would lead to reduced cell viability [10]. Nakano and his coworkers found that overproduction of ROS can be removed by adding melatonin into oocytes culture medium [40], and the development of oocytes could be improved [41, 42]. In the present study, the excessive ROS production in mouse oocytes due to cryopreservation could also be decreased by addition of melatonin to the culture medium. The mechanism of melatonin scavenging ROS is consistent with that of other antioxidants [43, 44].

Only when the melatonin concentration was within proper limits can it promote the development of oocyte and embryo either by scavenging the excessive ROS as described above, or by regulating gene expression through transcriptional factor Nrf2 [45]. Glutamate cysteine ligase modifier subunit (Gclm) could be regulated by Nrf2, and when expression of gene *Gclm* changes in oocytes, the GSH synthesis will be influenced. The decreased GSH level in oocytes [8] as well as the deficiency of the Nrf2 and Nrf1 transcription factors could result in early embryonic lethality [46]. In the present study, no significant change was observed in either the *Nrf2* expression or the GSH level after melatonin addition into the culture medium. Similarly the blastocyst rate of vitrified-warmed mouse oocytes after parthenogenetic activation was not affected by melatonin treatment.

However, when the melatonin concentration in the culture medium was beyond the proper limits, it may not show positive effect on the development of oocyte and embryo. In mouse, melatonin increased the IVF rate significantly at a concentration between 10^−6^ and 10^−4^ mol/L [47]; while at 10^−3^ mol/L, it significantly retarded the blastocyst rate [48]. In bovine, most effective melatonin concentrations ranged from 10^−9^ to 10^−7^ mol/L; while at 10^−5^ mol/L, it showed similar rates of cleavage and blastocyst to the control [49]. Similar results have been obtained in this study; when 10^−3^ mol/L melatonin was added into the culture medium, the cleavage rate of vitrified-warmed mouse mature oocytes after parthenogenetic activation, the expression of *Hsf1*, *Hsp90* and *Bcl-x1* was significantly decreased, but the blastocyst rate was similar to the control. In a word, it seemed that the melatonin has different effects on the development of embryos, depending on the concentrations [42, 50] and culture conditions [40, 51]. The length of time that oocytes were exposed to exogenous melatonin could also influence the development of embryos [27, 51]. Addition of melatonin into the medium in the whole process of culture, for instance, showed a positive effect on embryonic development in mice [47], sheep and goat [52, 53], pigs [27] and buffalo [54]. In the present study, the incubation time for culture of vitrified-warmed mouse oocytes in M_2_ medium with melatonin was only 1 h. In such a short period of time, the melatonin at the concentration range of 10^−5^-10^−9^ mol/L could not improve the rates of cleavage and blastocyst. It seemed that the culture time in M_2_ medium with melatonin should be prolonged.

## Conclusion

To sum up, ROS level was significantly decreased in 10^−3^ mol/L melatonin-treated group compared with the other concentration and groups, and the expression of *Hsp90aa1* increased significantly in 10^−7^ mol/L melatonin-treated group. GSH level, rates of cleavage and blastocyst development of oocytes after parthenogenetic activation and IVF were similar between the melatonin-treated and melatonin-free groups. Therefore, the addition of melatonin into the culture medium in the present study showed no positive effect on the subsequent development of vitrified-warmed mouse MII oocytes, even if there were alteration in gene expression.

## Abbreviations

10 % EG + 10 % DMSO: 10 % (v/v) ethylene glycol and 10 % (v/v) Dimethyl Sulphoxide in DPBS
EFS30: DPBS medium containing 30 % (v/v) ethylene glycol, 21 % (w/v) Ficoll 70, and 0.35 mol/L sucrose
IVF: *in vitro* fertilization
V: Volume
